# Silver Nanoplates for Colorimetric Determination of Xanthine in Human Plasma and in Fish Meat via Etching/Aggregation/Fusion Steps

**DOI:** 10.3390/s20205739

**Published:** 2020-10-09

**Authors:** Hung-Cheng Hsu, Pei-Wen Liao, Hsiang-Tzu Lee, Wei-Chen Liu, Mei-Lin Ho

**Affiliations:** Department of Chemistry, Soochow University, No. 70, LinShih Rd., Shih-Lin, Taipei 11102, Taiwan; cody11042@gmail.com (H.-C.H.); gigi2314565845@gmail.com (P.-W.L.); axiangli14@gmail.com (H.-T.L.); wei80915@gmail.com (W.-C.L.)

**Keywords:** silver nanoplates, colorimetric, Xanthine, fish, plasma

## Abstract

Silver nanoplates (AgP) were prepared and used in a colorimetric method for the evaluation of Xanthine (Xan) in blood plasma and fish meat. The detection mechanism for Xan was observed to occur via etching of AgP particles/aggregation/fusion steps, resulting in a color change from blue to grey. First, the basic Xan solution is adsorbed through partial substitution of capping molecules around the AgP with Xan, and then intermolecular hydrogen bonds form between AgP and AgP. Subsequently, the titrant Xan solution further etches the AgP and finally fuses particles together. Owing to the step by step mechanism, the response range towards Xan has two linear regression ranges: 0.15–0.60 μM and 0.61–3.00 μM, respectively. The detection limit in the range of 0.15–0.60 μM is 0.011 μM (S/N = 3). AgP exhibits good selectivity for Xan over other potential interferents such as amino acids and blood proteins. AgP achieves rapid detection of Xan and can be applied to the satisfactory determination of Xan in blood plasma and fish meat. This colorimetric sensor is easy to use, cost effective, fast, selective and user friendly.

## 1. Introduction

The quantitative determination of Xanthine (Xan) is important in the food industry and in clinical diagnosis [1]. In the food industry, after fish die, the supply of oxygen to the muscle tissue ceases due to the interrupted blood circulation, and anaerobic glycolysis remains the main way to produce energy (ATP) [2]. However, this energy demand exceeds the energy supply. ATP degradation in fish begins within about 6 h of death, and then degrades to a series of ATP-related compounds [3]. Xan is one the major metabolites in ATP degradation and increases during storage. Thus, Xan is an important indicator of chilled seafood freshness [4]. Recently, Xue et al. reported a fluorescence quenching method using ZnO nanomaterials to monitor Xan evolution in grass carp after post-mortem storage [5].

On the other hand, in clinical diagnosis, Xan is a metabolite of purine and is used as a marker of many diseases, such as gout, hyperuricemia [6], or renal failure [7]. Recently, Guo and co-workers used WO_3_ nanosheets as a peroxidase for colorimetric determination of Xan by combining the Xan oxidase and 3, 3′, 5, 5′-tetramethylbenzidine in urine [8]. In another work, Pu et al. used citrated-stabilized gold nanoparticles for the detection of traces of Xan. The method was based on the adsorption of the imide group of xanthine on gold nanoparticles and subsequently-induced particle aggregation [9]. A similar colorimetric method of detecting Xan using Xan oxidase and as-prepared bovine serum albumin to stabilize Au clusters in serum and urine was proposed by Wang et al. [10].

Silver nanoplates (AgP) are anisotropic metal nanoparticles and have attracted interest due to their morphology-dependent plasmonic properties and tunable plasmonic bands throughout the visible to near-infrared region [11,12]. In addition, owing to the Gibbs–Thomson effect, the sharp tips are more reactive than the edges, which are easily etched by other molecules, making them ideal materials for sensing applications [12,13]. Wang et al. reported a dual-read probe for colorimetric and fluorometric detection of uric acid-based AgP and carbon nanodots. AgP quenched the luminescence of carbon nanodots due to the inner-filter effect. In the presence of uric acid, etching by H_2_O_2_ is suppressed because the side facets of AgP are coated with uric acid, reducing the fluorescence of carbon nanodots [14].

Although the above noble metal nanoparticles have been used previously in colorimetric sensing for the detection of Xan, enzymes and other colorimetric agents need to be added to the reaction. Otherwise, the color-change response is time consuming upon addition of Xan. In the present work, we propose a simple, non-enzymatic and rapid colorimetric method for Xan detection in plasma and fish meat based on silver nanoplates alone.

## 2. Materials and Methods

### 2.1. Materials

Xanthine (C_5_H_4_N_4_O_2_, ≥99%), Sodium borohydride (NaBH_4_, ≥98%), Silver nitrate (AgNO_3_, ≥99.8%), Polyvinylpyrrolidone (PVP) and Hydrogen peroxide (H_2_O_2_, 34.5–36.5%) were purchased from Sigma-Aldrich, Saint Louis, MO, USA. Sodium citrate dihydrate (C_6_H_5_Na_3_O_7_·2H_2_O, ~99.8%) was purchased from J. T. Baker. L-Cysteine, L-Glutathione reduced, Threonine, Glutamine, L-Phenylalanine, Leucine, L-Lysine, Tryptophan, Methionine, Glycine, Alanine, Aspartic acid, Albumins, Globulins, Fibrinogen Caffeine, Theophylline and Theobromine were obtained from Sigma-Aldrich. Sodium dihydrogen phosphate (NaH_2_PO_4_·H_2_O, 99–102%) and Disodium hydrogen phosphate (Na_2_HPO_4_·2H_2_O, 98–102%) were purchased from Merck.

### 2.2. Instrument

The morphology and size of the AgP were characterized by transmission electron microscopy (TEM, JEOL model JEM-1230). Data on the absorption of the AgP were collected by ultraviolet-visible spectrometry (UV, HITACHI U-4100H). A refrigerated centrifuge (HITACHI CF15-RXII) was used to separate the AgP precipitate.

### 2.3. Preparation of Ag Nanoplates

The AgP were synthesized following the procedure of Mirkin et al. [15] with slight modification. Citrate aqueous solution (0.03 M, 6 mL) as the face-selective growth reagent was added to 100 mL of 0.01 M AgNO_3_ aqueous solution. PVP (0.7 mM, 6 mL) for surface passivation, which served as the size stabilizer, was added to the resulting solution. H_2_O_2_ (35%, 200 μL) was added into the solution as the shape-directing reagent. Finally, NaBH_4_ (0.1 M, 500 μL) was added to the solution as a reducing reagant [16]. After 15 min, the AgP were obtained.

### 2.4. The Optimization of PVP in the Synthesis of AgP

PVP–AgNO_3_ solutions with different molar ratios, i.e., 1:1, 3:1, 5:1, 7:1, 9:1 and 10:1 (mM:M), were prepared with different concentrations of PVP (0.1, 0.3, 0.5, 0.7, 0.9 and 1.0 mM) and 0.1 M AgNO_3_.

### 2.5. Analytical Procedures

The as-synthesized AgP was diluted in de-ionized water and the absorption value was adjusted to 2.8 for the characteristic experiment and 3.0 for the calibration curve and real sample test at 690 nm. The total volume was about 2.5 mL. The 10 μM stock solution of Xan was prepared in 30 mL NaOH aqueous solution and 70 mL PBS buffer solution at pH 7.0. For the titration experiment, 10 μL of Xan in varied concentrations was added to AgP solution. The absorption spectra of AgP in a quartz cell were recorded. The relative absorption changes (∆A) were calculated with Equation (1):

(1) $$\Delta A=\left|\frac{{\mathrm{Abs}}_{0,690\mathrm{nm}}-{\mathrm{Abs}}_{\mathrm{Xan},690\mathrm{nm}}}{{\mathrm{Abs}}_{0,690\mathrm{nm}}}\right|$$

where ${\mathrm{Abs}}_{0,690\mathrm{nm}}\;$ is the absorption value at 690 nm of AgP, i.e., without Xan, and ${\mathrm{Abs}}_{\mathrm{Xan},690\mathrm{nm}}$ is the absorption values upon the addition of different concentrations of Xan. The relative absorption changes (∆A) were calculated by the absorbance change before and after the addition of Xan.

### 2.6. Determination of Xan in Fish Meat

Largemouth bass were purchased from a local market, killed, and sectioned, and the pieces were stored at room temperature for different times, ranging from 1 to 5 days. The procedure of fish extraction was carried out in accordance with the method modified from Albelda and Li et al. [17,18]. Five g of fish meat was homogenized in 0.5 mL deionized water using a homogenizer (Nippi BioMasher^®^sp). The homogenate was centrifuged at 12,000 rpm for 10 min at room temperature, and the supernatant was then analyzed. A quantity of 40 μL of aqueous solution of NaOH (1 M) was added to AgP solution (2.5 mL). The supernatant (50 μL) was added to this AgP solution for subsequent analysis. The UV-Vis absorption spectra of the samples were scanned from 200 to 1300 nm. The concentration of Xan in fish meat was determined as described in the section “*Analytical procedures*”.

### 2.7. Effects of Interfering Compounds

To investigate the effects of other molecules on the absorbance of AgP solution, and thus the selectivity of this method, other potential interferents were also examined by the same procedure. Concentrations of each amino acid, i.e., L-Asparatic acid, Glycine, L-Tryptophan, L-Methionine, L-Lysine, L-Glutamine, L-Leucine, L-Threonine, L-Cysteine, L-Phenyalanine, L-Glutathione, and L-Alanine, Caffeine, Theophylline and Theobromine, were prepared as 1.5 mM. Ions and plasma proteins (i.e., 0.14 mM Sodium thiosulfate, 10 g/dL ppm Albumin, 5 g/dL Globulin and 1 g/dL Fibrin) were also prepared. After that, 0.57 μM Xan was added to a mixture of interferents and the spectra of the resulting solutions were measured.

### 2.8. Xan Determination in Human Plasma

Human blood plasma was measured using the newly-developed method and compared with the results from colorimetric Xan assay kits (Sigma-Aldrich). Human plasma (SRM 1950) was obtained from NIST (National Institute of Standards and Technology). A quantity of 40 μL of aqueous solution of NaOH (1 M) was mixed with AgP solution (2.5 mL) first. Then, 30 μL plasma was added into the AgP solution to measure the absorption change (see Equation (1)).

The assay kit allows Xan concentration analysis in human plasma, which is determined by an enzymatic reaction, and yields a colorimetric product at 570 nm. In the assay kits, Xan oxidase catalyzes Xan to uric acid and superoxide. The superoxide spontaneously degrades to H_2_O_2_, and in the presence of enzyme, reacts with dye reagent to generate the colorimetric product. Reagents used as the Xan standard solution and sample solution were 44 μL of Xan assay buffer, 2 μL of Xan enzyme mix solution, 2 μL of developer solution and 2 μL of peroxidase substrate solution. The blank solution used was 46 μL of Xan assay buffer, 2 μL of developer solution and 2 μL of peroxidase substrate solution. All the solutions mentioned above were mixed together and added to a 96 well plate (colorimetric reaction mixes). A total of 50 μL of different concentrations of Xan standards (0.5–4.5 μM) was prepared in Xan assay buffer and added to colorimetric reaction mixes with a final volume of 100 μL. The reaction was incubated for 30 min at room temperature, protected from light. Then, the absorbance was measured at 570 nm using the plate reader EPOCH^TM^. The calibration curve was obtained by subtracting the blank standard value from all readings. Typically, human plasma has Xan levels that fall above the linear detection range of this kit. Thus, the plasma was diluted 10 times prior to assay.

Continuous data are presented as mean ± SD. Pearson’s correlation coefficient, *r*, was calculated and a linear regression model was also used to assess the associations between continuous variables. All data were analyzed in software SPSS (Version 20.0, Chicago, IL, USA) and a *p* value of <0.05 was considered significant.

## 3. Results and Discussion

### 3.1. The Role of PVP Role in AgP Synthesis

**Scheme 1:**
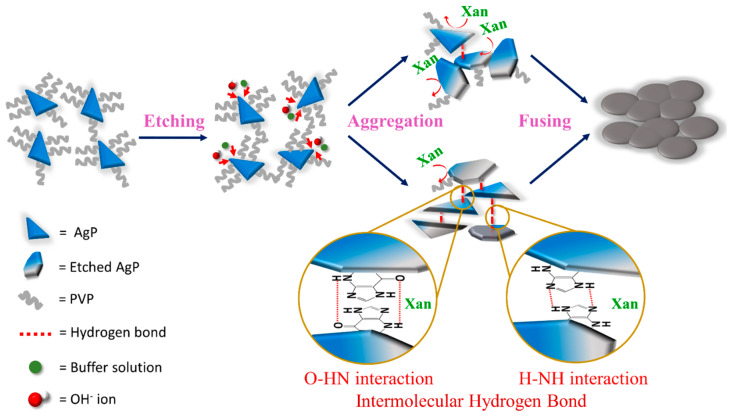
Schematic illustration of Xanthine (Xan) detection by etching/aggregation/fusion steps.

According to Skrabalak and co-workers, PVP can fulfill several roles in nanostructures, depending on the synthetic conditions [19]. For noble metals, PVP is adsorbed and stabilized on {100} Ag facets through the carbonyl group and nitrogen atom of the pyrrolidone ring via van der Waals attraction and direct binding through the oxygen atom (see Scheme 1) [19]. In addition, Pu et al. used an imide group, which has higher reactivity to bind to gold surfaces than amino and hydroxyl groups [9]. However, the use of anisotropic silver nanoplates with higher energy at the tips as well as a PVP coating with carbonyl and nitrogen atoms to react with the imide group has not been investigated. Thus, we propose a novel non-enzymatic colorimetric method for Xan detection.

**Figure 1:**
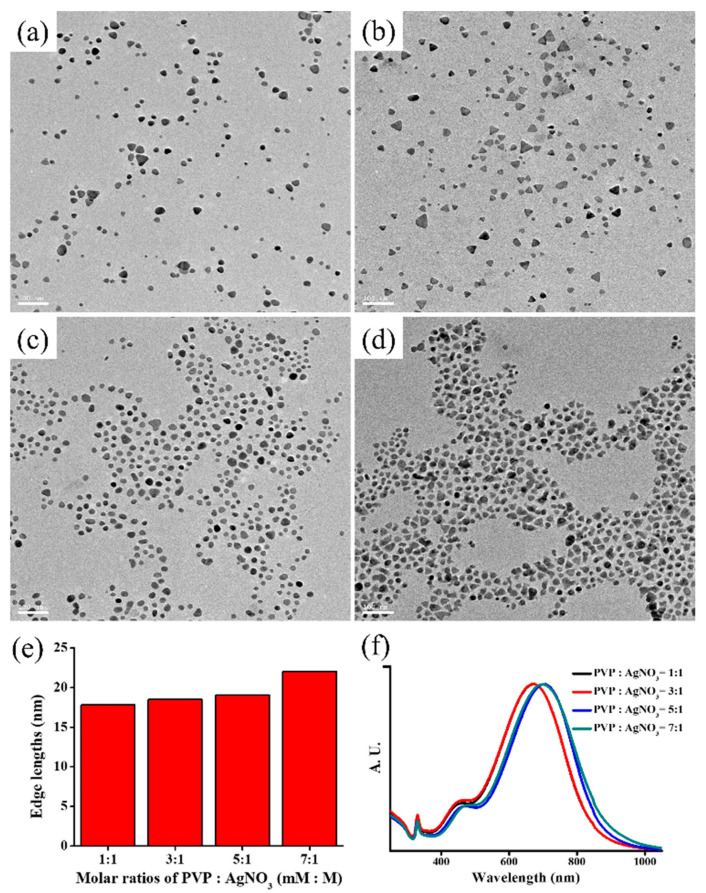
TEM images of silver nanoplates (AgP) prepared with various concentrations of Polyvinylpyrrolidone (PVP). The molar ratios of PVP–AgNO_3_ were 1:1, 3:1, 5:1 and 7:1 (mM:M) (**a**–**d**). The scale bar for each TEM image is 100 nm. (**e**) The edge lengths of AgP as a function of the molar ratio of PVP–AgNO_3_. (**f**) The UV-Vis spectrum of AgP synthesized with different molar ratios of PVP–AgNO_3_.

First, the molar ratios of the PVP–AgNO_3_ solutions were varied from 1:1 to 10:1 (mM:M). The differences in the TEM images and absorption spectra of AgP are presented in Figure 1a–d. As the concentration of PVP increased, the edge lengths of AgP increased (Figure 1e, and also Figure S1 in the supporting information). When the PVP increased, truncation of the corners was observed (Figure 1a–d). According to Xia et al., in the synthesis of AgP, the concentration of PVP will change the shape of AgP [20]. Regardless of the ratio of PVP to AgNO_3_, the characteristic absorption peaks appeared at 672–703 nm, 460–486 nm, and 331 nm, and were ascribed to the in-plane dipole mode, in-plane quadrupole mode, and out-of-plane quadrupole mode, respectively (Figure 1f). The characteristic absorptions of the in-plane dipole mode and in-plane quadrupole mode, especially for 460–486 nm, in UV-visible spectra of AgP were correlated to the edge lengths [21,22]. Simultaneously, the relative particle population of AgP peaked at a molar ratio of the PVP–AgNO_3_ solution of 7:1 (mM:M), which was chosen as the optimized synthesis condition for Xan sensing.

### 3.2. Sensing Principle for Xan

**Figure 2:**
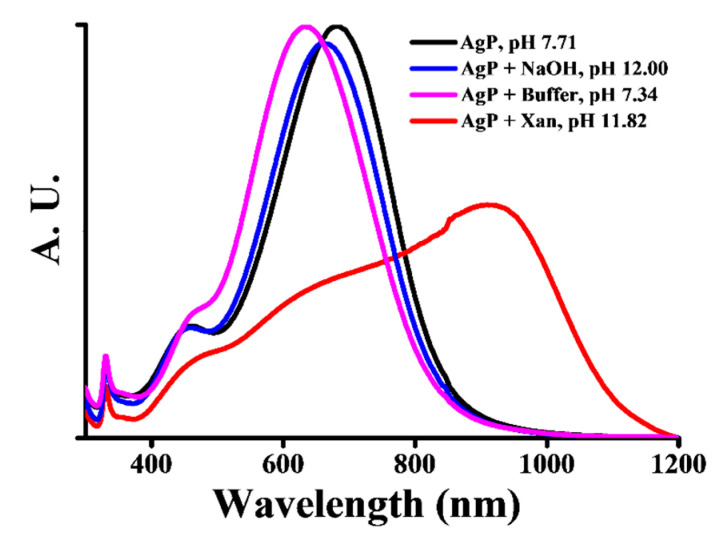
UV-Vis spectrum of AgP with addition of NaOH, buffer and Xan.

In our system, the formation of AgP and the process titration test (vide infra) were conducted under the dark condition. Hence, no light was involved in the synthesis and sensing procedure. Initially, plenty of citrate and a small portion of PVP capped to AgP increased the electrostatic repulsion force between the nanoparticles, and the AgP were dispersed in solution (also see Figure 1d). As shown in Figure 2, the peak intensity gradually decreased and the peak red-shifted with the addition of the Xan concentration (red line), implying that the size and morphology of the nanoparticles continuously changed. After exposure to the Xan solution, the out-of-plane quadrupole mode remained at 331 nm (Figure 2), indicating that Xan and OH^−^ did not affect the thickness of the AgP.

To clarify the role of OH^−^ and buffer solution in the basic Xan solution towards AgP, NaOH only and buffer solution were, respectively, added to the AgP solutions, and the pH values of the AgP solution at pH 7.71 changed to pH 12.0 and 7.34. The concentrations of NaOH and buffer were adjusted to the same ones in the titration of Xan. As shown in Figure 2, blue-shifts in the in-plane dipole mode in the absorption spectra were observed initially, implying that the AgP were influenced slightly when the OH^−^ and buffer solution were added. These blue-shifts would imply that the tips of the nanoplates become rounded. Yu et al. reported a similar result [23], but what causes the red-shift in absorption due to the addition of Xan in basic buffer solution?

**Figure 3:**
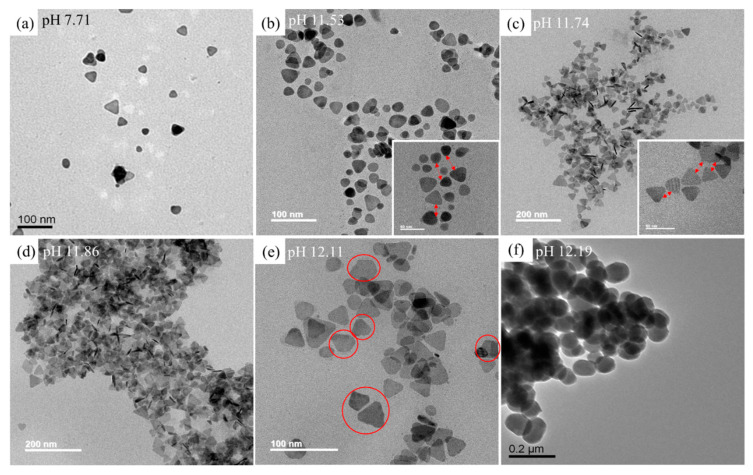
TEM images upon additions of different concentrations of Xan: (**a**) 0 μM, (**b**) 0.27 μM, (**c**) 0.57 μM, (**d**) 0.91 μM, (**e**) 2.86 μM and (**f**) 3.90 μM; and the respective pH values of individual conditions.

TEM images of AgP were captured after the addition of different concentrations of Xan, and the measured pH values of the individual resulting solutions are shown in Figure 3. From the TEM images, it appears that, as the basic Xan solution increased initially, the distance between AgP particles and AgP particles was reduced, the AgP tips were rounded (Figure 3a–c), and some AgP stacking gradually occurred (Figure 3c). Simultaneously, the absorbance intensity of the peak at 690 nm decreased, and the distances between AgP and AgP were reduced, which can be understood to indicate the replacement of surfactants and capping molecules around the AgP. Since the silver nanoplates were in the absence of surfactants, which have different surface free energy of σ{110} > σ{100} > σ{111} [23], theoretically, PVP could be used as a surfactant to stabilize the nanoparticles and preferentially cover the {110} facets [24]. When the OH^−^, buffer anions and Xan existed in the AgP solution, they would attach to the tips first and then replace some of the PVP around the AgP. Unlike in the study of Zhang et al., who described OH^−^ elevating the monodispersity of the AgP due to the increase in electrostatic repulsion force between AgP and elevation of the reducing ability of citrate [25], in our case, AgP gradually aggregated upon the addition of basic Xan to the AgP solution. In addition, a previous work of Pu et al. demonstrated that the imide group of Xan easily adsorbs onto the gold surface and then induces the aggregation of gold nanoparticles [9]. Therefore, we propose that basic Xan in AgP solution adsorbs onto the AgP surface, leading to the aggregation of the AgP particles [9].

When Xan was further introduced into this AgP system, the characteristic peaks of the in-plane mode in AgP gradually disappeared, the intensity decreased, and then the peak maximum gradually red-shifted. Figure 3c,d show the particles stacking substantially when the amount of Xan was increased. Since the in-plane dipole mode is very sensitive to the particle’s size change and the aspect ratio [22], the sizes of the AgP upon the addition of Xan were examined. From Figure 3a–d, with the stacking particles excluded, the average sizes of individual AgP particles were measured to be 22.5 ± 0.42, 19.0 ± 0.77, 46.7 ± 1.60, and 42.5 ± 0.23 (nm), respectively (Figure S2). These results suggested that AgP was etched by Xan at first (*vide supra*), and then the sizes of the resulting AgP nanoparticles become larger when more Xan was added to the AgP solution. The new absorption band at 800–1100 nm in the absorption spectra should be mainly ascribed to stacking-particles via aggregation. With the addition of Xan to the AgP, the exposed area of the {111} plane was much larger than that of {110} [24], and this can be rationalized by Xan preferentially reacting on the {111} plane and causing the stacking of AgP. If the Xan solution was increased further, i.e., the concentrations of OH^−^ and buffer anions were larger than the reducing reagent/surface stabilizer NaBH_4_ and citrate, the tips of AgP, marked in Figure 3e, were truncated; as the concentration of Xan in the cuvette exceeded 3.90 μM, the AgP fused together horizontally and finally transformed into large nanospheres (Figure 3f). A scheme of the possible morphological transformation mechanism for Xan detection is shown in Scheme 1.

**Figure 4:**
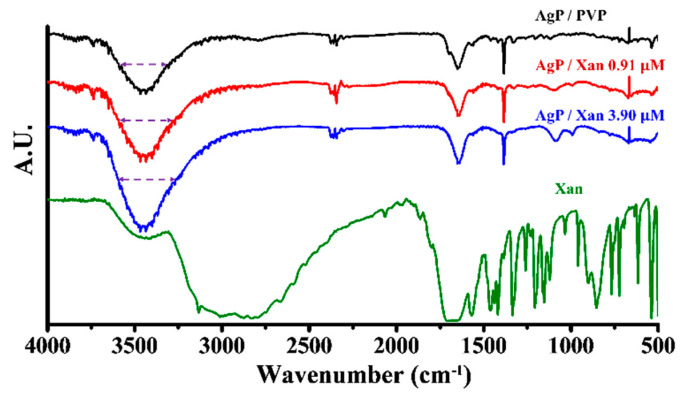
FTIR spectra of AgP/PVP, AgP with Xan added (AgP/Xan 0.91 μM, and AgP/Xan 3.90 μM), and free Xan.

FTIR measurements were performed to identify the interactions between AgP and Xan molecules. FTIR spectra of synthesized AgP capped with PVP (AgP/PVP), free Xan, and AgP with 0.91 μM and 3.90 μM of XAN added are depicted in Figure 4. In AgP/PVP, peaks were observed at 1645 and 3450 cm^−1^, confirming the –C=O and –OH stretching groups of PVP on the surfaces of AgP [19]. The vibrational bands of free Xan present at ~3100, 1674 (and 1568), 1232–1335, 1034–1206, 958, and 853 cm^−1^ were assigned to –NH, C=O, and C–N of the imidazole ring, C–N of the pyrimidine ring, N=C–H, and N–C–H of the imidazole ring vibrational stretching, respectively [26]. When AgP reacted with Xan (AgP/Xan), the disappearance of the broad band and some sharp peaks, such as those at ~3100, 1232–1335, 1034–1206, 958, and 853 cm^−1^, was accompanied by the appearance of small broad peaks at 992 and 1087 cm^−1^. Furthermore, the band at ~3450 cm^−1^ became broader upon increasing the addition of Xan, suggesting that H-NH stretching vibrations occurred due to the intermolecular hydrogen bonds between Xan and Xan. Furthermore, the peak at 1559 cm^−1^ corresponded to symmetry stretch vibration of C=O, and the peak intensity decreased after reaction with Xan. This vibration probably indicated hydrogen interactions of the O…N-H formation between Xan and Xan, and a further decrease in the C=O stretching. The characteristic peaks of PVP retained in AgP/Xan indicated that some PVP was still capped in AgP/Xan. The detection scheme for Xan is depicted in Scheme 1.

### 3.3. Absorption Titration Curve of Xan

**Figure 5:**
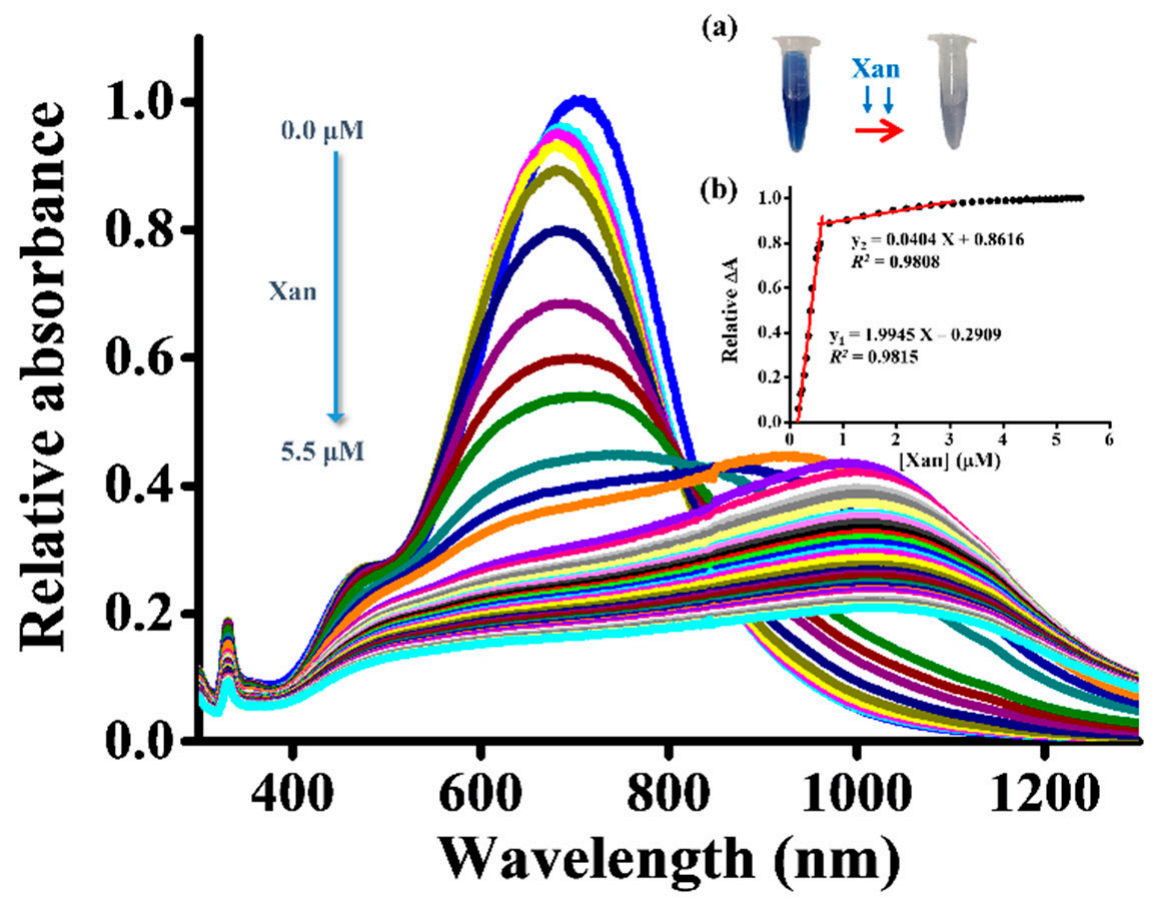
UV-Vis absorption spectra in the presence of different concentrations of Xan. Inset: (**a**) The colorimetric response upon addition of Xan; (**b**) the relationship between the absorbance change (ΔA) and the concentration of Xan.

Figure 5 shows the absorption spectrum of 2.5 mL AgP solution with the successive addition of 10 μM standard concentrations of Xan under optimal conditions. Upon addition of Xan, at basic buffer concentration, to AgP, the intensity of the peak of AgP at 690 nm gradually decreased and a new peak appeared in the near infrared region. The absorbance values were recorded at 690 nm to calculate the absorbance changes (ΔA, as shown in Equation (1) in the Materials and Methods section). Simultaneously, the color of the solution changed immediately from blue to blue-purple upon exposure to Xan. Finally, the peak shifted to ~1100 nm; concurrently, the final color of the solution turned grey (Figure 5a). As shown in Figure 5a, all of the color changes could be observed with the naked eye.

The ratio of change in absorbance before and after the addition of Xan was plotted against the concentration of Xan, and two linear response ranges, 0.15–0.60 μM and 0.61–3.00 μM, were obtained. As shown in Figure 5b, when the Xan concentration was increased to the range of 0.15–3.00 μM, the value of ΔA increased. The linear regression equations of Xan detection were ΔA = 1.9945 [Xan] − 0.2909 for 0.15–0.60 μM (R^2^ = 0.9815) and ΔA = 0.0404 [Xan] + 0.8616 for 0.61–3.00 μM (R^2^ = 0.9808), respectively. The detection limit of Xan was calculated to be 0.011 μM (S/N = 3). The analytical performance of the AgP is compared with those of other materials in Table 1. As shown in Table 1, the response range of AgP met the requirements for Xan detection in clinical and food industry monitoring.

In addition, the titration curve had two linear ranges, one at lower concentration and one at higher concentration. This can be understood from the differences in the TEM images. At lower concentrations, i.e., <0.60 μM, the titrant OH^−^, buffer, and Xan adhered to the individual AgP particles and partially replaced the surfactant around the AgP. When the concentration of titrant was elevated, i.e., >0.61 μM, the intermolecular hydrogen bonds that formed between Xan molecules caused the large-scale stacking of AgP particles via aggregation, etching and fusion steps. These two environments and the free energy of the systems in these two ranges were different and hence resulted in two different response ranges. To challenge the present material for food industry and clinical use, fish meat was analyzed after 1–5 days of storage at room temperature, and human plasma was subsequently tested.

### 3.4. Xan Determination in Fish Meat

**Figure 6:**
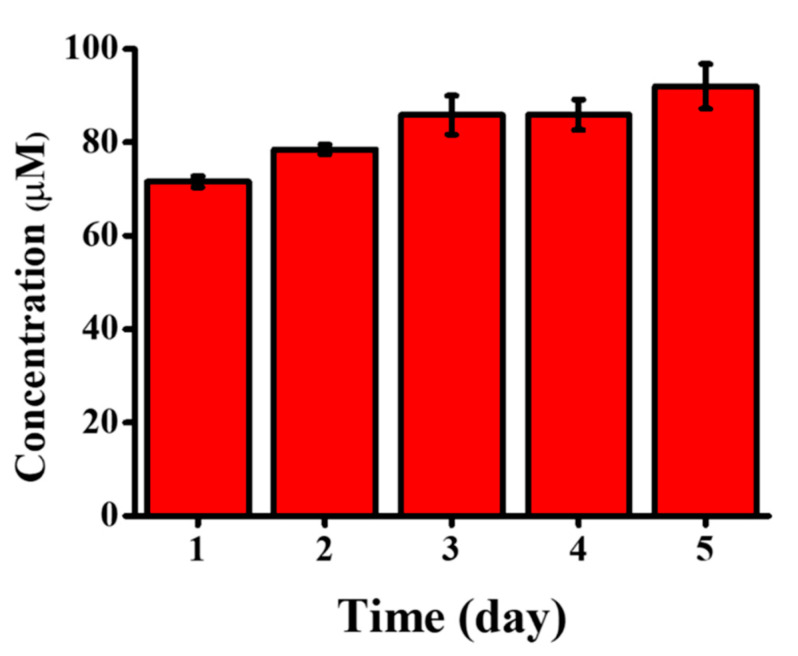
The Xan evolution in largemouth bass over 5 days of storage at 30 °C.

To verify the applicability of AgP in the food industry, the present method was used to determine the Xan concentrations in 5 g samples of fish meat stored at 4 °C and room temperature (30 °C), respectively, for durations of 1 to 5 days. We found that if the fish meat of the largemouth bass was stored at 4 °C, the Xan content was below the detection limit of our present method in the first 3 days, and the values did not change. In contrast, in the fish meat stored at 30 °C, as shown in Figure 6, the concentration of Xan increased with storage times from 1 to 5 days. The pre-treatment of fish meat is about 30 min. From the measured absorbance changes, the Xan content in 5 g of fish meat could be deduced during storage. The Xan level increased from 71.5 ± 1.22 to 92.0 ± 4.79 during storage. In comparison with amperometric measurements, the Xan mass content in fish meat matched those reported by Dervisevic et al. [4] and Devi et al. [1].

### 3.5. Effects of Interfering Compounds in Plasma

The selectivity of the AgP for the detection of Xan in plasma was then estimated. The effects of coexisting substances in plasma were investigated. Known concentrations (1.5 mM) of amino acids and blood proteins were prepared at higher than normal levels in plasma, at least 5–80 fold higher than those of healthy individuals (see Table S1 and Figure S3), respectively. To these were added AgP solution, and the absorbance change (ΔA) was calculated. As shown in the inset of Figure S3, the color changed with the additions of different interferents. It has been shown that most amino acids have no interfering effects on the analysis of Xan, but compounds in the thiol-containing group, e.g., L-Cysteine, L-Glutathione, and sodium thiosulfate, which have stronger affinity to AgP [30], slightly interfere in the analysis of Xan. Zare et al. have reported that L-phenylalanine can act as a reducing agent to cap gold nanoparticles [31]. In the present method, L-phenylalanine and L-alanine produced slight interference in the determination of Xan. When 0.57 μM Xan solution was added to a mixture of potential interferents (mix), i.e., excess concentrations of interferents higher than normal levels in healthy individuals, the increase in absorbance was apparent.

Owing to the similar structures, Xan alkaloids, including caffeine, theophylline, and theobromine, were also tested. As shown in Figure S4, N-methyl derivatives (caffeine, theophylline, and theobromine) have no obvious interfering effects on the analysis of Xan.

### 3.6. Xan Determination in Human Plasma

An increased plasma level of Xan is a criterion of tissue hypoxia in clinical diagnosis. The normal Xan level in plasma should be between 0.5 and 2.5 μM [6]. In a study by Pleskacova et al., plasma samples were obtained from 15 healthy volunteers, and Xan levels were analyzed by the HPLC method. A triplicate analysis revealed that the Xan mean level was 4.31 μM [32]. The present method was applied to the analysis of human plasma from NIST. As far as we know, no reports in the literature are focused on Xan levels in plasma. The Xan levels were determined by our present method and also compared to those obtained using a commercial colorimetric assay kit (Table 2). All data were measured in triplicate. The Xan levels were found to be 5.47 ± 0.49 μM by our method and 5.76 ± 0.54 μM by the colorimetric method. The value of relative standard deviation with our method was within 10%. The values obtained from the two distinct methods were very close to reported values in normal plasma [32]. A *t*-test performed at a 95% confidence level demonstrated that the results obtained by the two methods did not differ significantly. Matrix effects were evaluated and recoveries were determined with spiking of four different Xan concentrations, and the mean values were within ± 10% of 100%, supporting the acceptable accuracy of the method. Based on the results in Table 2, the Xan levels detected by the proposed method (*X*) and the conventional kit (*Y*) were significantly correlated, with a Pearson correlation of *r* = 0.997 (*p* < 0.001). In addition, in a linear regression analysis, the Xan levels measured using the two methods were significantly associated (*p* < 0.001) in a fitted regression line (equation *Y* = 3.820 + 0.869*X*).

## 4. Conclusions

In this study, we proposed an enzyme-free, low-cost, rapid response and colorimetric method to detect Xanthine (Xan) in fish meat after post-mortem storage and in human plasma. Homogeneous silver nanoplates (AgP) were successfully prepared. The PVP concentration had a great influence on the size and population of the nanoparticles produced. The Xan detection of AgP was investigated by TEM, UV-VIS and FTIR analysis. The detection mechanism is proposed to be as follows: Xan molecules, OH^−^ and buffer anions adsorb onto the surface of AgP and displace citrate and PVP molecules, inducing AgP aggregation through the intermolecular hydrogen bonds. The solution further etches AgP and finally fuses the AgP together, and an absorption shift follows. The solution color changes from purple to grey in correlation with the concentration of Xan. The detection limit is 0.011 μM. The Xan in fish meat was measured after a period of 5 days of storage. Furthermore, the AgP was also successfully applied to determine the Xan levels in human plasma, and good agreement with those obtained by commercial assay kit was found. To the best of our knowledge, few works have focused on combining colorimetric methods with nanoparticles to measure Xan in fish meat or in plasma. The sensing method of the system described herein can be applied for the detection of Xan in clinical diagnosis and the food industry.

## Figures and Tables

**Table 1:** Comparison of different colorimetric sensors for detection of Xan.

Material	Linear Range (μM)	Limit of Detection (μM)	Ref.
**BSA−Au clusters ^a^**	1.0−200	0.5	[11]
**Se−g−C_3_N_4_^b^**	0.16–40	0.016	[27]
**AuNPs ^c^**	0.125–6.0	0.023	[9]
**BSA−DTT−CuNCs ^d^ (nanoclusters)**	0.50–100	0.38	[28]
**WO_3_ nanosheets−TMB ^e^**	25–200	1.24	[8]
**Hemin@CDs ^f^**	0.17−33	0.11	[29]
**AgP**	0.15–0.60 0.61–3.00	0.001	This work

^a,d^ BSA = Bovine serum albumin. ^b^ Se−g−C_3_N_4_ = Selenium doped graphite phase carbon nitride. ^c^ AuNPs = Gold nanoparticles. ^d^ DTT = Dithiothreitol, CuNCs = Copper nanoclusters. ^e^ TMB = 3, 3′, 5, 5′-tetramethylbenzidine. ^f^ Hemin = Heme−proteins, CDs = Carbon dots.

**Table 2:** Determination of Xan levels in human plasma.

Xan Added (M) ^b^	Commercial Assay Kit (M) ^a^	Our Proposed Method (M)	Recovery (%)	R.S.D. (%) ^c^
0.0	5.76 ± 0.54	5.47 ± 0.49	/	8.96
31.3	33.7 ± 3.98	33.8 ± 2.47	107.9	7.30
61.5	56.8 ± 6.96	59.6 ± 5.93	97.0	9.94
80.0	80.2 ± 4.97	82.4 ± 5.51	103.0	6.68
123.0	111.7 ± 10.56	128.3 ± 11.1	104.3	8.66

^a^ Colorimetric method. ^b^ “Xan found” was equal to the value of subtracting “our proposed method” from “Xan measured”. ^c^ R.S.D.: relative standard deviation.

## Supplementary Materials

The following are available online at https://www.mdpi.com/1424-8220/20/20/5739/s1, Figure S1: (a–f) histogram analysis for the edge length of the silver nanoplates in PVP–AgNO_3_ in molar ratios of 1:1, 3:1, 5:1, 7:1, 9:1 and 10:1 (mM:M). Figure S2: TEM spectra, particle size distribution of silver nanoplates under exposure to different concentrations of xanthine: (a) 0.0, (b) 0.27, (c) 0.57 and (d) 0.91 μM. Note that the stacking particles were excluded. Figure S3: Relative absorbance value (∆A) and a photographic image (inset) of the AgP in the absence and presence of different species. Mix denotes the mixture of all interferents. Figure S4: Relative absorbance value (∆A) of the AgP in the presence of Xan and caffeine, theophylline, and theobromine. Table S1: The concentrations of interferents in healthy human plasma.
