# Supplementary material for: Silver Nanoplates for Colorimetric Determination of Xanthine in Human Plasma and in Fish Meat via Etching/Aggregation/Fusion Steps
Source: Sensors (Basel). 2020 Oct 9;20(20):5739. doi: 10.3390/s20205739 (PMC7599804; doi:10.3390/s20205739)
Supplement: Supplementary file 1 [file sensors-20-05739-s001.pdf]

Supplementary materials:

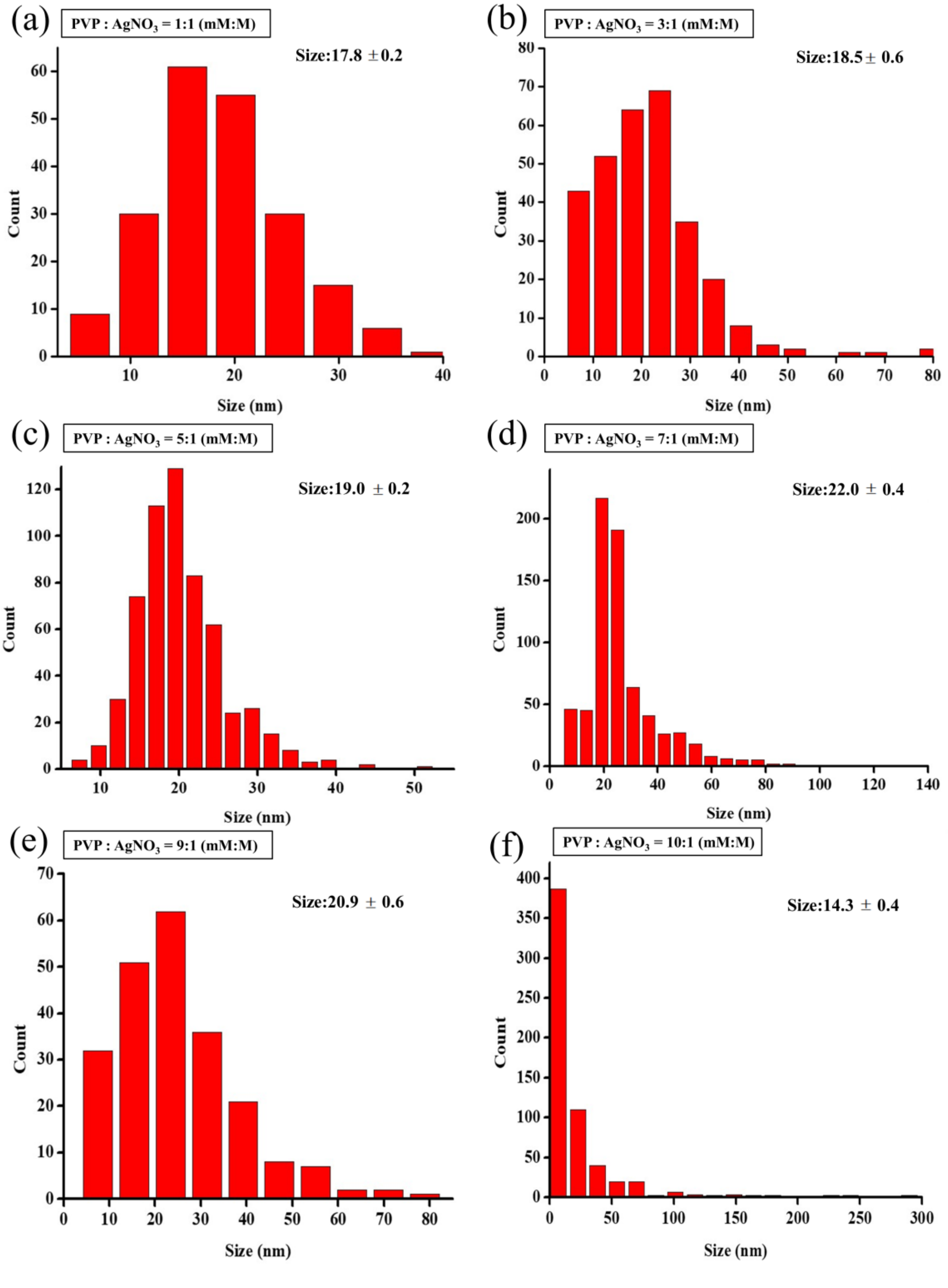

Figure 1. (**a**)-(**f**) histogram analysis for the edge length of the silver nanoplates in PVP–$AgNO_3$ in molar ratios of 1:1, 3:1, 5:1, 7:1, 9:1 and 10:1 (mM:M).

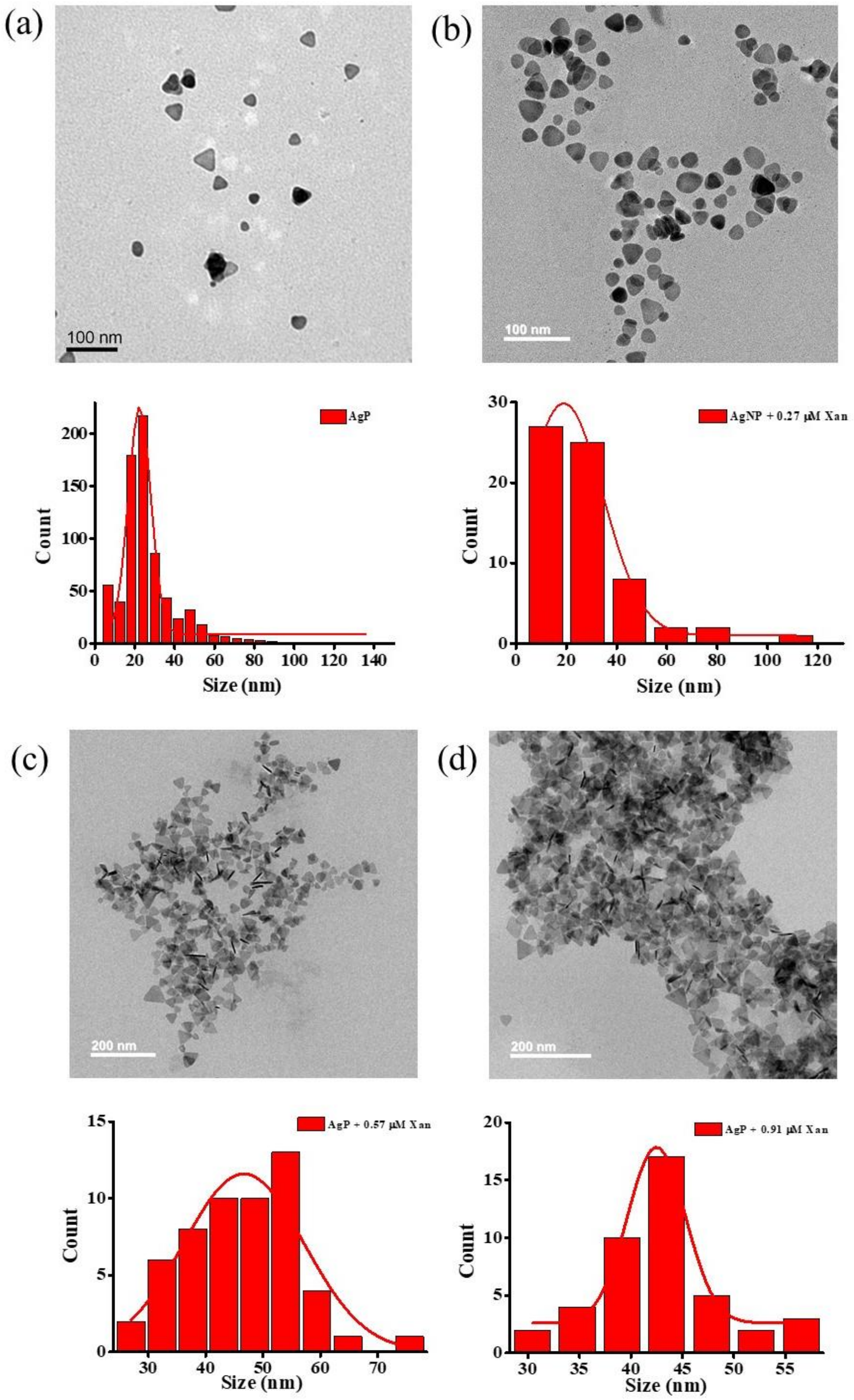

Sample number	Additive	Size (nm)
a		22.47 ± 0.42
b	0.27 μM Xan	19.01 ± 0.77
c	0.57 μM Xan	46.65 ± 1.60
d	0.91 μM Xan	42.48 ± 0.23

Figure 2. The TEM spectra, the particle size distribution of silver nanoplates under exposure of different concentrations of xanthine: (**a**) 0.0, (**b**) 0.27, (**c**) 0.57 and (**d**) 0.91 μM. Note the stacking particles were excluded.

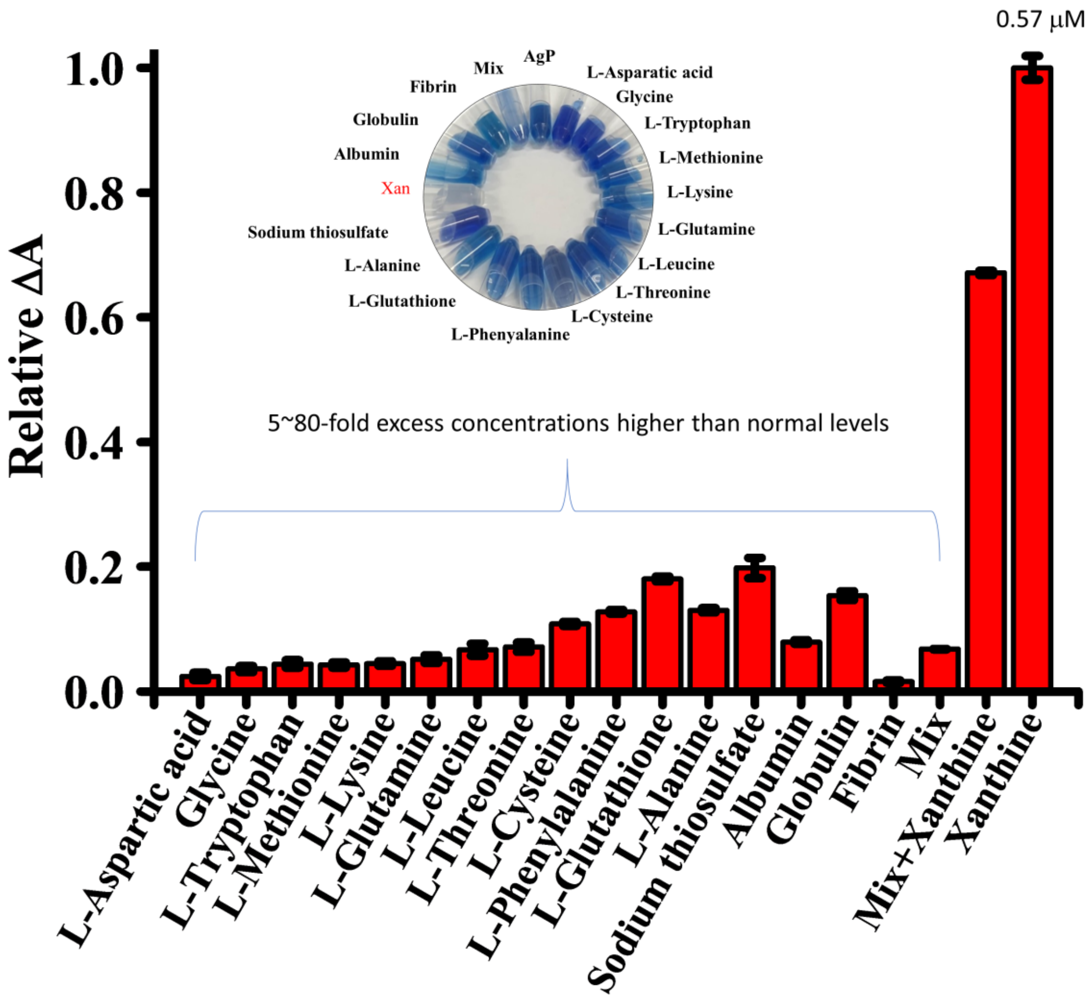

Figure 3. Relative absorbance value (ΔA) and a photographic image (inset) of the AgP in the absence and presence of different species. Mix denotes the mixture of all interferents. .

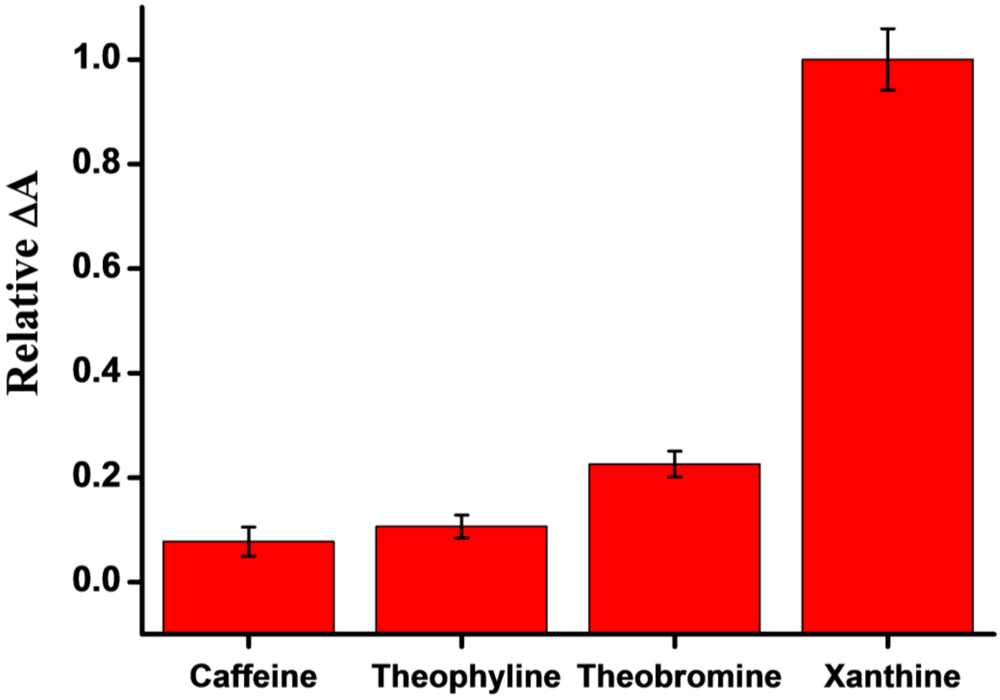

Figure 4. Relative absorbance value (ΔA) of the AgP in the presence of Xan and caffeine, theophylline, and theobromine. .

Table 1. The concentractions of interferents in healthy human plasma.

Compound	Healthy individual	The test in our method	Reference
**L-Aspartic acid**	0.021 mM	1.5 mM	3
**Glycine**	0.325 mM	1.5 mM	3
**L-tryptophan**	0.056 mM	1.5 mM	2
**L-Methionine**	0.029 mM	1.5 mM	2
**L-Lysine**	0.127 mM	1.5 mM	2
**L-Glutamine**	0.042 mM	1.5 mM	3
**L-Leucine**	0.099 mM	1.5 mM	3
**L-Threonine**	0.128 mM	1.5 mM	3
**L-Cysteine**	0.034 mM	1.5 mM	3
**L-Phenylalanine**	0.056 mM	1.5 mM	2
**L-Glutathione**	0.34±0.11 μM	1.5 mM	5
**L-Alanine**	0.5 mM	1.5 mM	3
**Sodium thiosulfate**	11.3±0.11 mM	0.14 mM	6
**Albumin**	3.5-5.0 g/dL	10 g/dL	4
**Globulin**		5 g/dL	
**Fibrin**		1 g/dL	
**Xanthine**	0.5-2.5 μM	0.57 μM	1

References:

1. Kalimuthu, P.; Leimkuhler, S.; Bernhardt, P.V. Low-Potential Amperometric enzyme biosensor for xanthine and hypoxanthine. *Anal. Chem.* **2012**, *84*, 10359–10365.
2. Jiye, A.; Trygg, J.; Gullberg, J.; Johansson, A.I.; Jonsson, P.; Antti, H.; Marklund, S.L.; Moritz, T. Extraction and GC/MS analysis of the human blood plasma metabolome. *Anal. Chem.* **2005**, *77*, 8086–8094.
3. Psychogios, N.; Hau, D.D.; Peng, J.; Guo, A.C.; Mandal, R.; Bouatra, S.; Sinelnikov, I.; Krishnamurthy, R.; Eisner, R.; Gautam, B.; Young, N.; Xia, J.; Knox, C.; Dong, E.; Huang, P.; Hollander, Z.; Pedersen, T.L.; Smith, S.R.; Bamforth, F.; Greiner, R.; McManus, B.; Newman, J.W.; Goodfriend, T.; Wishart, D.S. The human serum metabolome. *PLoS ONE* **2011**, ***6***, Article e16957.
4. Parodi, A.; Miao, J.; Soond, S.M.; Rudzinska. M.; Zamyatnin, A.A. Albumin nanovectors in cancer therapy and imaging. *Biomolecules*. **2019**, *9*, 218–241.
5. Wendel, A.; Cikryt, P. The level and half-life of glutathione in human plasma. *FEBS Letters.* **1980**, *120*, 209–211.
6. Ivankovich, A.D.; Braverman, B.; Stephens, T.S.; Shulman, M.; Heyman, H.J. Sodium thiosulfate disposition in humansrelation to sodium nitroprusside toxicity. *Anesthesiology* **1983**, *58*, 11–17.
